# Mechanisms Applied by Protein Inhibitors to Inhibit Cysteine Proteases

**DOI:** 10.3390/ijms22030997

**Published:** 2021-01-20

**Authors:** Livija Tušar, Aleksandra Usenik, Boris Turk, Dušan Turk

**Affiliations:** 1 Department of Biochemistry and Molecular and Structural Biology, Jozef Stefan Institute, Jamova cesta 39, 1000 Ljubljana, Slovenia; livija.tusar@ijs.si (L.T.); aleksandra.usenik@ijs.si (A.U.); boris.turk@ijs.si (B.T.); 2 Centre of Excellence for Integrated Approaches in Chemistry and Biology of Proteins (CIPKeBiP), Jamova cesta 39, 1000 Ljubljana, Slovenia; 3 Faculty of Chemistry, University of Ljubljana, Večna pot 113, 1000 Ljubljana, Slovenia; 4 Institute of Regenerative Medicine, I.M. Sechenov First Moscow State Medical University, Bol’shaya Pirogovskaya Ulitsa, 19c1, 119146 Moscow, Russia

**Keywords:** mechanisms of inhibition, cysteine proteases inhibitors, structural-based inhibition, compiled kinetic data

## Abstract

Protein inhibitors of proteases are an important tool of nature to regulate and control proteolysis in living organisms under physiological and pathological conditions. In this review, we analyzed the mechanisms of inhibition of cysteine proteases on the basis of structural information and compiled kinetic data. The gathered structural data indicate that the protein fold is not a major obstacle for the evolution of a protease inhibitor. It appears that nature can convert almost any starting fold into an inhibitor of a protease. In addition, there appears to be no general rule governing the inhibitory mechanism. The structural data make it clear that the “lock and key” mechanism is a historical concept with limited validity. However, the analysis suggests that the shape of the active site cleft of proteases imposes some restraints. When the S1 binding site is shaped as a pocket buried in the structure of protease, inhibitors can apply substrate-like binding mechanisms. In contrast, when the S1 binding site is in part exposed to solvent, the substrate-like inhibition cannot be employed. It appears that all proteases, with the exception of papain-like proteases, belong to the first group of proteases. Finally, we show a number of examples and provide hints on how to engineer protein inhibitors.

## 1. Introduction

Previously, we reviewed cysteine protease protein inhibitors and their role in regulation of proteolysis [1]. In the review, we classified the inhibitors according to their physiological roles using quantitative criteria of enzyme kinetics, the delay time of inhibition, and the stability time of inhibition, established by Joseph Bieth in the 1980s [2,3]. Delay time, d(t), is the time needed to achieve ≈99% of inhibition (d(t) = ln 2/I_o_ × k_ass_), and roughly equals seven half-lives of the reaction. In this equation, I_o_ represents the physiological concentration of the inhibitor and k_ass_ is an approximation of the association rate constant. The stability time of reversible inhibitors is defined as the minimal time in which the EI (E, enzyme; I, inhibitor) complex remains undissociated (t(s) = ln 2/k_diss_), where k_diss_ represents the dissociation rate constant. This suggest that inhibitors were of physiological relevance when the delay time was below 1 s and, for reversible inhibitors, when the stability time was above 10 min [2]. Two types of inhibitors were introduced, emergency and regulatory [1,4]. Emergency inhibitors rapidly trap a protease and maintain it in a stable complex preventing any undesired activity. Regulatory inhibitors, by comparison, modulate the protease activity under physiological conditions. They can be further divided into threshold, buffer, delay, and pro-inhibitor sub-types. The threshold-type inhibitors prevent undesired protease activation. The buffer-type inhibitors reversibly and rapidly bind proteases, and when their physiological substrate appears, they also rapidly release them and thereby prevent undesired and potentially harmful proteolysis in the absence of their substrate. The delay-type inhibitors irreversibly (or pseudo-irreversibly) and slowly bind their target, thereby enabling proteolysis for a limited amount of time, whereas pro-inhibitors require initial processing by a protease to become inhibitory. Detailed kinetic studies in which K_ass_ and K_diss_ are measured are seldomly performed. To gain insight into the relative differences between various protease inhibitor interactions, we rely predominantly on their ratio, Ki. This link between biochemical principles of inhibition and physiology does not require an update, however, the determination of a number of new structures of cysteine protease inhibitors indicates that canonical mechanisms of inhibition should be updated [5]. Because no review, including ours, completely covers a broad topic such as protein inhibitors of cysteine proteases, we want to remind readers that other related reviews have been undertaken that describe various aspects of cysteine protease inhibition [6,7,8,9,10,11,12,13,14].

Cysteine proteases use the reactive site cysteine as the catalytic nucleophile and the histidine to perform peptide bond hydrolysis. In MEROPS [15], an online database that provides an insight into peptidases, there are 16 clans of cysteine peptidases and some that are unclassified, of which four among them include proteases with mixed catalytic types. They are further divided into 97 families of structurally and sequentially related peptidases, of which 18 families belong to the four clans of mixed catalytic types. Activity of many of these is regulated by protein inhibitors, which are either endogenous or originate from the invading organisms [15]. The MEROPS list of clans and families of protein inhibitors of proteases contains 27 clans and about four times as many structurally and sequentially related families. Their classification has little relation to the type of protease they target; inhibitors such as macrocypins, thyropins, and serpins can simultaneously bind two different families of cysteine proteases with their two distinct reactive sites. Table 1, Table 2, Table 3, Table 4 and Table 5 present the available structures of the complexes between protein inhibitors and cysteine proteases, including their family classification, Protein Data Bank (PDB) codes [16], and publication references. Table A1 shows the binding and kinetic constants K_i_ for their interaction with target proteases to provide an experimental basis for their classification. Due to the differences in K_i_ values, the same inhibitor can belong to several types in respect to the protease they inhibit. For example, cystatins differentiate among exo- and endo-peptidases, and the inhibitory fragment of the p41 form of the invariant chain associated with the major histocompatibility class II molecule (the p41 fragment) can be, in respect to the target, emergency and buffer inhibitors, and also the delay type and pro-inhibitor (a detailed explanation and references are provided below). For our review of the mechanisms of inhibition of cysteine proteases, we selected crystal structures of diverse types of inhibitors, which either target large groups of related proteases, such as papain-like proteases and caspases, or others including calpastatin and securin, with unique mechanism(s) of inhibition.

## 2. Inhibitors of Papain-Like Cysteine Proteases

Papain-like cysteine proteases are the largest family (C1 according to MEROPS) among the cysteine proteases, and likely the most studied. The subgroup of cysteine cathepsins is involved in a myriad of physiological functions from protein turnover to processing of antigens, hormones, and bone remodeling [11]. Moreover, the crystal structure of papain was among the first enzyme structures determined [17], and Schechter and Berger introduced the nomenclature of substrate binding sites and positioning of the substrate when studying papain interaction with a polyalanine peptide [18]. Papain-like cysteine proteases are inhibited by several groups of protein inhibitors that are involved in the regulation of physiological and pathogenic conditions. In this section, we present cystatins as the largest group of inhibitors [7,10], followed by falstatins, chagasins, thyropins, clitocypins and macrocypins, and staphostatins inhibiting a papain-related protease staphopain from family C47. Serpins are an important group of inhibitors of serine and cysteine proteases, including papain-like cysteine proteases. To the best of our knowledge, the structure of their complex with a representative of cysteine proteases is still lacking. Thus, we include a brief overview of their mechanism in Section 3.4.

### 2.1. Cystatins

**Figure 1:**
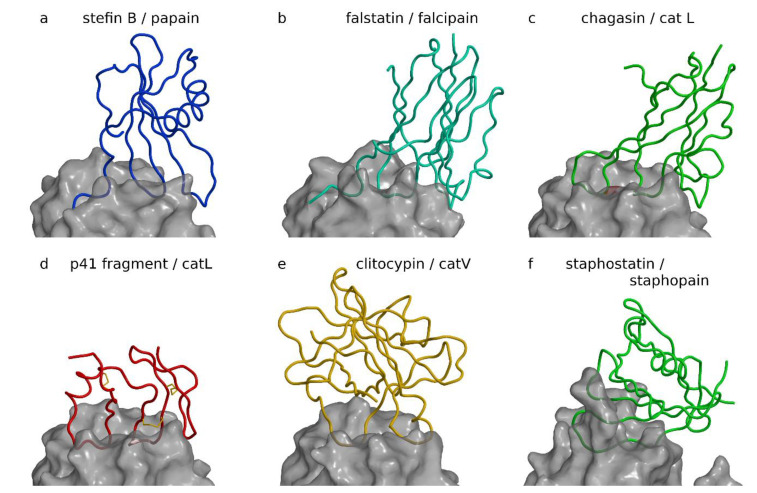
Inhibitors of papain-like and related proteases. Complexes are shown with the same view across the active site cleft and the same scale after superimposition of proteases to cathepsin L in the p41 fragment complex. Figure was prepared using MAIN [35] and rendered with Raster3d [36]. (**a**) Stefin B papain complex ([21], PDB code 1STF). The stefin B chain is shown as a blue coil on the semitransparent background of the white surface of papain. (**b**) Inhibitor of cysteine protease (ICP) (falstatin) falcipain complex ([37], PDB code 3PNR). ICP, also known as falstatin from *Plasmodium berghei,* is shown as a cyan coil on the semitransparent background of the white surface of falcipain-2. (**c**) Chagasin cathepsin L complex ([38], PDB code 2NQD). The chagasin chain is shown as a green coil on the semitransparent background of the white surface of cathepsin L. (**d**) p41 fragment cathepsin L complex ([39], PDB code 1ICF). p41 fragment chain shown as a red coil on the semitransparent background of the white surface of cathepsin L. The three disulfide bonds of the p41 fragment are shown as yellow sticks. (**e**) Clitocypin cathepsin V complex ([40], PDB code 3H6S). The clitocypin chain is shown as a yellow coil on the semitransparent background of the white surface of cathepsin V. (**f**) Staphostatin staphopain complex ([41], PDB code 1PXV). The staphostatin chain is shown as a green coil on the semitransparent background of the white surface of staphopain.

Cystatins were the first discovered and are the best studied endogenous inhibitors of cysteine cathepsins [19]. Their major function appears to be protection of the organism from undesired endogenous proteases; however, they also protect against invading microorganisms and parasites, which apply cysteine proteases to invade the host. Cystatins are divided into three families: the stefins, the cystatins, and the kininogens. Stefins and cystatins are single-domain proteins, whereas kininogens contain three cystatin domain repeats. The cystatin fold was revealed by the crystal structure of chicken cystatin ([20], PDB code 1CEW), which provided the basis for the elephant trunk model of their interaction with papain-like cysteine proteases. The model was later confirmed by the crystal structure of stefin B in complex with papain ([21], PDB code 1STF), shown in Figure 1a. Cystatins block the reactive site with the N-terminal trunk and a loop. The positions of the N-terminal trunk and the loop in the structure are stabilized by a β-sheet, which is at the concave side stabilized by an α-helix. The second loop of cystatins interacts with the active site cleft and contributes to the binding [22], however, it is not directly involved in blocking the access to the cysteine histidine pair of reactive site residues. They bind to their targets in a two-step mechanism, with the loops providing the initial binding and the N-terminus locking the complex and strengthening the interaction [23], indicating that the inhibitor undergoes a conformational change on binding. The structure of the complex showed that cystatins do not interact with the reactive site of the target protease in a substrate-like manner; hence, cystatins are not slowly degrading substrates, in contrast to at the time most studied inhibitors of serine proteases, such as bovine pancreatic trypsin inhibitor (BPTI) ([24], PDB code 2TGP). Cystatins reversibly bind papain-like cysteine endopeptidases in the nM to fM range [22,25,26,27,28], whereas cysteine exopeptidases are inhibited in the mM to nM range [29,30,31,32]. Nevertheless, they bind to cathepsins B and H, as demonstrated by the crystal structure of their complexes ([33], PDB code 3K9M; [34], PDB code 1NB5). Due to the span in the binding constants, stefins and cystatins are emergency and buffer inhibitors. Table 1 shows complexes for the cystatin family.

**Table 1:** List of the available structures of the complexes between protein inhibitors and cysteine proteases: cystatins.

	Protein Inhibitor			Cysteine Protease			
MEROPS ID	Name	Organism	MEROPS ID	Name	Organism	PDB ID	Reference
I25	**Cystatin** **family**						
I25.001	stefin A	*Homo sapiens*	C01.060	cathepsin B	*Homo sapiens*	3K9M	[33]
I25.001	stefin A	*Homo sapiens*	C01.040	cathepsin H	*Sus scrofa*	1NB3/1NB5	[34]
I25.001	stefin A	*Homo sapiens*	C01.032	cathepsin L	*Homo sapiens*	3KSE	
I25.001	stefin A	*Homo sapiens*	C01.009	cathepsin V	*Homo sapiens*	3KFQ	
I25.003	stefin B	*Homo sapiens*	C01.001	papain	*Carica papaya*	1STF	[21]
I25.006	cystatin E/M (cystatin 6)	*Homo sapiens*	C13.004	legumain, animal-type	*Homo sapiens*	4N6N	[42]
I25.011	ovocystatin	*Gallus gallus*	C01.046	falcipain-2	*Plasmodium falciparum* 3D7	1YVB	[43]
I25.014	tarocystatin	*Colocasia esculenta*	C01.001	papain	*Carica papaya*	3IMA	
I25.014	CTD of tarocystatin	*Colocasia esculenta*	C01.001	papain	*Carica papaya*	3LFY	

### 2.2. Falstatins

Falstatins, which are inhibitors of cysteine proteases (ICPs) from *Plasmodium* spp., as demonstrated by the crystal structure of a falcipain 2 complex ([37], PDB code 3PNR, Table 2), seemingly use the same interaction pattern to inhibit their target cysteine protease falcipain as cystatins—the N-terminal peptide interacts with the protease surface in a similar manner to stefin B, followed by a two-loop arrangement filling the active site (Figure 1b). This is the extent of the similarity, however, because the *Plasmodium* spp. ICPs are based on a β-sandwich related to the immunoglobulin fold, and the apparent N-terminal trunk is a partially disordered loop, which thereby lacks structure, whereas the part in contact with the target protease is ordered. Hence, ICPs from *Plasmodium* spp. utilize a three-loop arrangement to block the active site of papain-like proteases. The visible interaction loop is the second loop and longer than the first interaction loop in cystatins because it must span the space between the two β-sheets in the fold. ICPs from *Plasmodium* spp. bind non-selectively, and bind to papain-like and related cysteine proteases in the pM to nM range [44]. Falstatin does not inhibit cysteine proteases with exopeptidase activity (cathepsins B and C), and proteases of other catalytic classes, such as serine proteases (trypsin and chymotrypsin), aspartic proteases (pepsin and renin), and metalloproteases (collagenase and matrix metalloprotease-2). Falstatins are also supposed to inhibit calpain-1 in the sub-nM range, and caspases-3 and -8 in the nM range [44], yet these results were later disputed by Hansen et al. [37].

**Table 2:** List of the available structures of the complexes between protein inhibitors and cysteine proteases: falstatin, chagasins, thyropin, clitocypin, and staphostatins.

	Protein Inhibitor			Cysteine Protease			
MEROPS ID	Name	Organism	MEROPS ID	Name	Organism	PDB ID	References
I71	**Falstatin family**						
I71.001	falstatin (PbICP-C)	*Plasmodium berghei*	C01.046	falcipain-2	*Plasmodium falciparum* 3D7	3PNR	[37]
I42	**Chagasin family**						
I42.001	chagasin	*Trypanosoma brucei*	C01.001	papain	*Carica papaya*	2CIO	[45]
I42.001	chagasin	*Trypanosoma cruzi*	C01.001	papain	*Carica papaya*	3E1Z	[46]
I42.001	chagasin	*Trypanosoma cruzi*	C01.046	falcipain-2	*Plasmodium falciparum* 3D7	2OUL	[32]
I42.001	chagasin	*Trypanosoma cruzi*	C01.060	cathepsin B	*Homo sapiens*	3CBJ/3CBK	[47]
I42.001	chagasin	*Trypanosoma cruzi*	C01.032	cathepsin L	*Homo sapiens*	2NQD	[38]
I31	**Thyropin family**						
I31.002	MHC II invariant chain p41 form	*Homo sapiens*	C01.032	cathepsin L	*Homo sapiens*	1ICF	[39]
I48	**Clitocypin family**						
I48.001	clitocypin	*Clitocybe nebularis*	C01.009	cathepsin V	*Homo sapiens*	3H6S	[40,48,49]
I57	**Staphostatin** **family**						
I57.001	staphostatin B	*Staphylococcus aureus*	C47.002	staphopain B	*Staphylococcus aureus*	1PXV	[41]
I57.001	staphostatin B	*Staphylococcus aureus*	C47.002	staphopain B	*Staphylococcus aureus*	1Y4H	[50]

### 2.3. Chagasins

Chagasins are endogenous inhibitors of papain-like cysteine proteases from parasites such as *Trypanosoma cruzi* and *Leishmainia mexicana*. They have a similar fold as that of ICPs from *Plasmodium* spp. In Figure 1c, the structure of the complex of chagasin from *Trypanosoma cruzi* with cathepsin L is shown ([38], PDB code 2NQD, Table 2). The N-terminal trunk has been replaced by a loop, hence their interaction with the active site is based on a three-loop arrangement. Several complexes of chagasin with other cysteine cathepsins ([47], PDB code 3CBK, Table 2), papain ([46], PDB code 3E1Z; [45], PDB code 2CIO, Table 2), and falcipain-2 ([32], PDB code 2OUL, Table 2) have been reported. Chagasin is a nonspecific inhibitor of papain-like proteases, with K_i_ values in the pM to 100 nM range [32,38]. Several structures of complexes of chagasin with other cysteine proteases, cathepsin B ([47], PDB code 3CBK, Table 2), papain ([46], PDB code 3E1Z, Table 2), and falcipain 2 ([32], PDB code 2OUL, Table 2) have been reported. Endogenous physiological inhibition of cruzipain by chagasin is reversible and tight-binding with a Ki value in the pM range [51].

### 2.4. p41 Fragment

The p41 fragment sequence is embedded in the much larger invariant chain associated with the major histocompatibility complex (MHC) class II-associated p41 invariant chain fragment. The p41 fragment is homologous to sequential repeats, called thryoglobulin type-1 domains, due to their numerous occurrences in thyrogobulin [52,53]. The inhibitors with this sequential repeat are called thyropins [54]. The crystal structure of the p41 fragment in a complex with cathepsin L ([39], PDB code 1ICF, Table 2) revealed its fold, which is stabilized by three disulfide bonds (Figure 1d). The CWCV sequence, the signature of the fold, is at its core, and with which two disulfide bonds stabilize the three stranded β-sheet. The third disulfide bond attaches the helix to the body of the fold. The p41 fragment is the smallest of the cysteine protease inhibitors composed of only 64 amino acid residues. Similarly to inhibitors of cysteine proteases (ICPs) and chagasin, it uses a three-loop arrangement to bind to the active site. The first and the second loops of the p41 fragment occlude the reactive site, whereas the third forms additional contacts at the primed side of the active site cleft. The p41 fragment is more selective than cystatins. It inhibits the endopeptidases cathepsins L and V in the pM range, cathepsins K and F in the nM range, and cathepsin S in the µM range, but does not inhibit exopeptidases [55,56]. On the basis of these values and its concentration, we can consider the p41 fragment an emergency inhibitor (cathepsins L and V) or a buffer-type inhibitor (cathepsin S) [56]. It may even be possible that its inhibitory role is activated after processing of the invariant chain, hence the p41 fragment may also be a pro-inhibitor or a delay-type inhibitor. In addition to thyroglobulin, several other proteins contain this structural motif, such as saxiphilin, which binds saxitoxin, a toxin from bullfrogs [57]. However, the inhibitory function has been confirmed for few of these, including equistatin inhibiting cysteine cathepsins [58]; testicans-2 and -3, which inhibit matrix metalloproteases and serine proteases’ saxiphilin, which inhibits papain and cathepsin L; and the inhibitor from salmon egg, which inhibits papain and cysteine cathepsins [58,59,60,61,62,63,64]. In other proteins, such as nidogen, insulin growth factor-binding proteins, and the human carcinoma marker protein GA733 (also called TROP2 [65]), the inhibitory role has not been demonstrated, although it has been suggested that their thyroglobulin type-1 domains may serve as a buffer for the activity of endosomal proteases during thyroglobulin processing [66,67,68]. Hence, the conclusion that the thyroglobulin type-1 repeat is a structural motif occasionally employed as an inhibitor of proteases still applies [69].

### 2.5. Clitocypins and Macrocypins

Clitocypins and macrocypins from mushroom species (basidiomycetes) *Clitocybe nebularis* and *Macrolepiota procera*, respectively, are primarily inhibiting papain-like cysteine proteases, although inhibition of legumain and serine proteases has also been observed [49]. This property is due to their β-trefoil fold, the hallmark of Kunitz-type inhibitors, which are the classical serine protease inhibitors ([40], PDB code 3H6S, Table 2). The β-trefoil fold has a remarkably simple core composed of a sixfold β-barrel and six exposed loops stabilized by short β-antiparallel strand arrangements (reviewed in [48]). The crystal structure of the complex between clitocypin and human cathepsin V (Figure 1e) shows that clitocypin (and similar macrocypins) binds in the active site of papain-like proteases with two broad loops occluding the catalytic site residues from both sides of the active site cleft. Two broad loops are a common denominator in all complexes presented in Figure 1 and indicate convergence in the mechanism of inhibiting papain-like proteases. An exception is the staphostatin/staphopain complex shown in Figure 1f, in which the protease staphopain is not papain-like, but a papain-fold-related protease. Clitocypin and macrocypins inhibit papain-like proteases in the 10 pm to 100 nM range [49,70].

### 2.6. Staphostatins A and B

Staphostatins A and B are endogenous inhibitors of the secreted cysteine proteases from *Staphylococcus aureus*, staphopains A and B, which are remotely related to cysteine cathepsins. Each staphostatin specifically inhibits its target staphopain. Staphostatins have a β-barrel fold, which is similar to that of lipopains and different from that of cystatins [71]. The crystal structure of staphostatin B in complex with the staphopain B catalytic site mutant C243A ([41], PDB code 1PXV; [50], PDB code 1Y4H, Table 2) shows that staphostatin B binds in the active site cleft of staphopain in a substrate-like manner with the sequence IGTS mimicking the P2 to P2′ substrate residues (Figure 1f), which explains why staphostatins are slowly degraded substrates. Filipek et al. [50] further showed that the G98 residue is crucial for inhibitory activity because its mutations in other residues converted staphostatin B to a significantly better substrate of staphopain B. The extended conformation of the G98-T99 section is stabilized by a hydrogen-bonding ladder with the antiparallel positioned S93-S92-T91 section in the central β-sheet, likely keeping the G98-T99 peptide bond at a distance to prevent its hydrolyses. Hence, rather than a loop, an antiparallel β-sheet hydrogen bonding ladder stabilizes the bound conformation.

### 2.7. Serpins

Serpins obtained their name from their ability to inhibit serine proteases, however, they are cross-class inhibitors that also inhibit cysteine proteases such as cathepsins, calpains, and caspases. With over 1500 representatives in Archaea, Prokarya, and Eukarya, in addition to a number of viruses, serpins are the largest and most widely dispersed family of peptidase inhibitors [72], and include 37 human serpins [73]. Squamous cell carcinoma antigen 1 (SCCA1 also called serpin B3) is an epithelial-derived serpin that inhibits the endopeptidases cathepsins K, L, and S [74,75]. Heparin was also found to enhance the interaction with the target proteases, similar to the interaction of several serpins with plasma serine proteases [76]. In addition to SCCA1, cathepsin L was shown to be inhibited by the cross-class inhibitor endopin 2C [77], which preferentially inhibits cathepsin L over papain and elastase.

Among cathepsins, cathepsin L is specifically targeted with another cross-class serpin, hurpin (serpin B13) [78]. Another cross class inhibitor SRP-6 was shown to inhibit cathepsins K and L, and calpain-2 [79]. Although serpins are primarily endogenous inhibitors, they are also employed as part of a defense against pathogens. SCCA1 was shown to inhibit staphopains [80] and falcipain-2 [81], whereas SCCA 2 was shown to inhibit mite allergen cysteine protease Der p1 [82]. Inhibition of caspases by serpins is described below.

## 3. Inhibitors of Caspases

**Figure 2:**
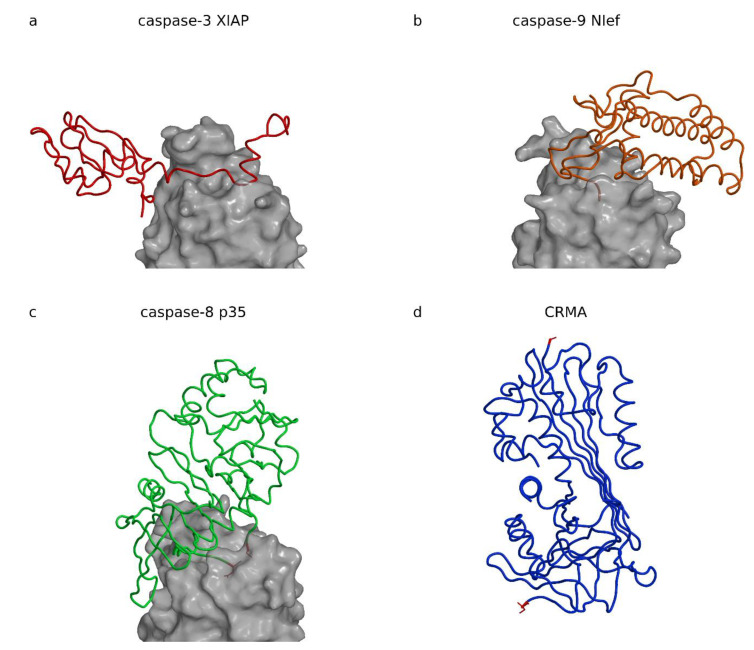
Inhibitors of caspases. Complexes are shown with the same view and scale aligned after superimposition of the caspases to the caspase-3 in the complex with X-linked inhibitor of apoptosis (XIAP). Figure was prepared using MAIN [35] and rendered with Raster3d [36]. (**a**) Human XIAP caspase-3 complex ([83], PDB code 1I3O). The XIAP chain is shown as a red coil on the semitransparent background of the white surface of caspase-3. (**b**) *Escherichia coli* Nlef caspase-9 complex ([84], PDB code 3V3K). The Nlef chain is shown as an orange coil on the semitransparent background of the white surface of caspase-9. (**c**) Baculovirus p35 caspase-8 complex ([85], PDB code 1I4E). The p35 chain is shown as a blue coil on the semitransparent background of the white surface of caspase-8. (**d**) The CrmA chain ([86], PDB code 1F0C) is shown as a blue coil with the cleaved residues A359 and S359A shown as stick model in red.

Cell apoptosis is an important mechanism during embryogenesis and organism growth to make place for new cells and tissues. Later in life, apoptosis is required for removal of defected, infected, and malicious cells, and is crucial for organism survival. Caspases take a central role in the apoptosis initiation and execution phases. They are cysteine proteases belonging to their own C14 family (MEROPS: [15]). Because removal of infected cells by apoptosis is also a defense mechanism against infections, it is no coincidence that caspase inhibitors have been found in cell invaders such as viruses (baculovirus inhibitor of apoptosis proteins (IAPs) and protein p35, cowpox virus serpin cytokine response modifier A (CrmA)) and bacteria (*Escherichia coli* effector protein Nlef). Figure 2 shows structures of four caspase inhibitors, three in complexes made with X-linked inhibitor of apoptosis (XIAP), Nlef, and p35, and the serpin CrmA. As Figure 2 demonstrates, the folds of these four inhibitors have no common structural motif, which suggests their common role or at least an evolutionary relationship. In addition, the view in which all three caspases are superimposed shows that XIAP, Nlef, and p35 are positioned at positions most widely spread from left to right in respect to the active site of their target caspases.

### 3.1. XIAP

**Figure 3:**
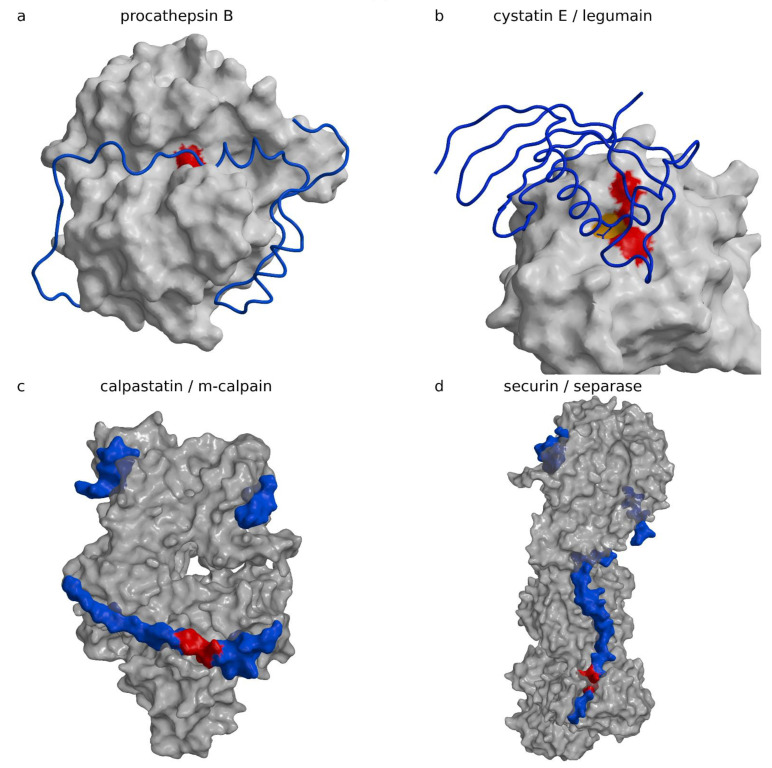
Other inhibitors. Complexes of inhibitors are shown in views and scales adjusted to each complex and size. Figure was prepared using MAIN [35] and rendered with Raster3d [36]. (**a**) Procathepsin B ([98], PDB code 3PBH). The chain of cathepsin B propeptide is shown as a blue coil on the white surface of mature enzyme part of the structure. The surface part corresponding to the catalytic pair of C29 H199 residues is colored red. (**b**) Cystain E legumain complex ([42], PDB code 4N6N). Cystatin E is shown as a blue ribbon, with the P1 residue N39 side chain bound in to the legumain S1 site shown as a red stick model. Legumain is shown as a white surface with the S1 binding pocket colored orange and the part corresponding to the reactive site residues C189 H148 colored red. (**c**) Calpastatin m-calpain complex ([99], PDB code 3DF0). Calpastatin is shown as a blue surface with the loop out region indicating the position above the reactive site of calpain shown in red. Calpain-m is shown as a semitransparent white surface. (**d**) Securin separase complex ([100], PDB code 5ULS, 5ULT). Securin is shown as a blue surface with the region from 262 to 265 bound above the reactive site of separase shown in red. Securin is shown as a semitransparent white surface.

XIAP belongs to the protein family of inhibitor of apoptosis proteins (IAPs) present in viruses [87,88] and eukaryotes. IAPs activity is embedded in the baculoviral IAP repeat (BIR) domains, the homologues of which are present throughout all eucaryotic kingdoms [89]. They are involved in regulation of the activity of executioner and initiator caspases-3, -7, and -9. The crystal structures of XIAP (BIR domain 2) in complex with caspase-7 ([90]; PDB code 1I51, Table 3); [91]; PDB code 1I4O, Table 3) or caspase-3 ([83]; PDB code 1I3O, Table 3) revealed that XIAP binds along the active site cleft of the caspase (Figure 3a).

**Table 3:** List of the available structures of the complexes between protein inhibitors and cysteine proteases: IAPs, p35, and NIef.

	Protein Inhibitor			Cysteine Protease			
MEROPS ID	Name	Organism	MEROPS ID	Name	Organism	PDB ID	Reference
I32	**IAP family**						
I32.002	cIAP-BIR3	*Homo sapiens*	C14.010	caspase-9 (Nter pept)	*Homo sapiens*	3D9T	[92]
I32.004	XIAP	*Homo sapiens*	C14.004	caspase-7	*Homo sapiens*	1I4O	[93]
I32.004	XIAP	*Homo sapiens*	C14.004	caspase-7	*Homo sapiens*	1I51	[90]
I32.004	XIAP-BIR2	*Homo sapiens*	C14.004	caspase-7	*Homo sapiens*	1KMC	
I32.004	XIAP-BIR2	*Homo sapiens*	C14.003	caspase-3	*Homo sapiens*	1I3O	[83]
I32.004	XIAP-BIR3	*Homo sapiens*	C14.010	caspase-9	*Homo sapiens*	1NW9	[94]
I32.009	DIAP1	*Drosophila melanogaster*	C14.019	caspase Dronc (pept)	*Drosophila melanogaster*	1Q4Q	[95]
I32.009	DIAP1-BIR1	*Drosophila melanogaster*	C14.015	drICE	*Drosophila melanogaster*	3SIP	[96]
I50	**Baculovirus p35 family**						
I50A	p35	*Autographa californica nucleopolyhedrovirus*	C14.009	caspase-8	*Homo sapiens*	1I4E	[85]
I50A	p35	*Autographa californica nucleopolyhedrovirus*	C14.009	caspase-8	*Homo sapiens*	2FUN	[97]
	**Family I94**						
I94.001	NIeF	*Escherichia coli*	C14.010	caspase-9	*Homo sapiens*	3V3K	[84]

XIAP has a short helical region the authors called a “hook” followed by a long linker, which runs along the active site cleft of the caspase and the terminal BIR2 domain with the “sinker” interacting with the S4 pocket.

The “hook” and the “sinker” with the BIR2 domain attach to the caspase surface and stretch the linker. The linker runs along the active site cleft in the direction opposite to the substrate binding, thereby precluding its cleavage. The concept is reminiscent of cysteine cathepsin inhibition by their propeptides in their zymogen form (Figure 3a shows procathepsin B; [98], PDB code 3PBH, Table 4), in particular, the smallest one of cathepsin X ([101], PDB code 1DEU, Table 4). The similarity is dual: (i) the propeptides of cysteine cathepsins run along the active site cleft in the direction opposite to the substrate, and (ii) the propeptides form smaller and larger domains, which all begin with a helix positioned at the prime side of the active site cleft approximately above the reactive site cysteine histidine pair. Because prodomains of cysteine cathepsins and BIR2 lie on opposite sides of the active site cleft, the similarity reflects a convergent solution. The fold of the BIR domain is, interestingly, not important for inhibition, as noted earlier [1]. An important contribution of the XIAP BIR2 domain to a two-site interaction inhibition of caspases-3 and -7 has been proposed by Scott et al. [102], in which the weak interaction of the linker sequence that inhibits activity must be stabilized by the binding of the BIR2 domain surface groove that binds caspase-7 at a site exposed only during the maturation cleavage. IAP proteins inhibit caspases by several distinct mechanisms. For example, the BIR3 domain of XIAP inhibits caspase-9 by blocking the dimerization of the catalytically-inactive monomers that is required for activity ([94], PDB code 1NW9).

**Table 4:** List of the available structures of the complexes between protein inhibitors and cysteine proteases: proenzymes.

	Zymogens			
MEROPS ID	Name	Organism	PDB ID	References
C10.001	exotoxin B (streptopain)	*Streptococcus pyogenes*	1DKI	[103]
C01.060	procathepsin B	*Homo sapiens*	3PBH	[98,104]
C25.003	gingipain RgpB	*Porphyromonas gingivalis W83*	4IEF	[105]
C01.032	procathepsin L	*Homo sapiens*	1CJL/1CS8	[106]
C01.001	propapain	*Carica papaya*	3TNX	[107]
C01.003	procaricain	*Carica papaya*	1PCI	[108]
C01.013	procathepsin X	*Homo sapiens*	1DEU	[101]
C01.060	procathepsin B	*Rattus norvegicus*	1MIR	[109]
C01.036	procathepsin K	*Homo sapiens*	7PCK	[110]
C01.036	procathepsin K	*Homo sapiens*	1BY8	[111]
C01.034	procathepsin S	*Homo sapiens*	2C0Y	[112]
C01.040	procathepsin H	*Homo sapiens*	6CZK/6CZS	[113]

### 3.2. Escherichia coli Effector Protein Nlef

*Escherichia coli* effector protein Nlef inhibits caspases-4, -8, and -9 [84,114,115,116,117,118]. The crystal structure of the complex between Nlef and human caspase-9 indicates two Nlef segments interacting with the active site cleft of caspase-9, the protein C-terminal sequences L196, Q197, C198, and G199; and the H145, H146, and S157 (Figure 2b) ([84], PDB code 3V3K, Table 3). The Nlef can be considered a substrate analog, only falling short of one residue to be cleaved. Instead, its C-terminal residue lacks the side chain and deploys the C-terminal carboxylic group of G199 to mimic the aspartate at the P1 position. This concept is reminiscent of the cathepsin C exclusion domain-binding mechanism, which provides the N-terminal carboxylic group of the aspartic residue to block access in the active site beyond the S2 site, and thereby restricts activity of cathepsin C to a di-amino-peptidase [119]. The similarity is even more striking in the case of a metalloprotease inhibitor from *Erwinia chrisantemi*, which fills half of the active site with its N-terminus and thereby blocks access to substrates [120]. Interestingly, it was shown that Nlef is only one among many *Escherichia coli* effector proteins causing delay and inhibition of apoptosis. Its role appears minor due to its low expression profile, however, when over expressed it can severely impact apoptosis [84].

### 3.3. Baculovirus Protein P35

Baculovirus protein p35 is a broad-spectrum caspase inhibitor. It has a flexible reactive loop with the caspase recognition sequence DQMD ([121]; PDB code 1P35). The crystal structure of the p35 in complex with caspase-8 ([85], PDB code 1I4E, Table 3) shows how the cleaved loop remains trapped in its covalent attachment to the enzyme with D87 forming the thioester bond to the caspase-8 C360 reactive site cysteine and D84 bound to the S4 binding site. The covalent interaction explains why the crystal structure of the complex shows loose packing of the p35 chains entering the active site cleft (Figure 2c). These two chains belong to two different N-termini sequentially far apart: the D87 at the N-terminus resulting from the p35 cleavage and the repositioned residue C2 at the p35 N-terminus. Later, the sulfhydril group of C2 was shown to be crucial for preventing hydrolysis of the caspase C360p35 D87 thioester bond by trapping it in the exchange with the p35 thioester bond of C2D87, as demonstrated by Lu et al. ([97], PDB code 2FUN, Table 3), who found that the N-terminal fragment of p35 appeared as a circular peptide after dissociation from the complex.

### 3.4. Cowpox Virus CrmA

The cowpox virus CrmA structure was chosen to represent serpins as inhibitors of caspases. Its crystal structure was determined in the cleaved form (Figure 2d), with the P1 and P1′ residues A359 and S359A, respectively, more than 60 Å apart ([86], PDB code 1F0C; [122], PDB code 1M93). At conditions preventing hydrolysis, it was shown that serpins’ reactive site loop binds in the active site cleft of trypsin in extended conformation ([123], PDB code 1K9O), which is in a strong contrast with the cleaved form structure. The mechanism of inhibition of cysteine proteases was not demonstrated with a crystal structure of a complex of the cleaved form, however, the typical serpin insertion of the reactive site sequence in the central β-sheet suggests the trypsin-like mechanism of inhibition ([124], PDB code 1EZX), and formation of a thiol ester with the catalytic cysteine that in part unfolds the target protease [125,126,127]. CrmA is a minimal serpin. It targets caspases-1 with Ki in the pM range [128,129] and caspase-8 in the sub-nM range [130,131,132], but poorly inhibits executioner caspases-3, -6, and -7 [131,132], and probably caspase-10 [130]. Similar to a number of other serpins, such as serpin B9 [133] and myxoma virus serpin serp2 [130], it also inhibits serine protease granzyme B [130]. Although serpins eventually separate from their target protease, they cannot bind back to it. Serpins are suicide substrates irreversibly changed upon reaction. In fact, the pathway, called the suicide substrate branched pathway mechanism, is even more complicated because it involves one two-way and four one-way processes, all of which end in separation of serpin from its target (reviewed in [12]). Therefore, in such cases K_i_ does not apply. For simple comparison of inhibition rates of serpins, we advise the use the kinetic constant k_ass_ of the first step only.

## 4. Some Other Types of Inhibitors

### 4.1. Propeptides of Papain-Like Cysteine Proteases

Propeptides of papain-like cysteine proteases are in their essence inhibitors attached to the framework of the mature protease structure [134,135]. They are not entirely specific to their cognate enzyme and may inhibit other enzymes in the family [136]. All propeptides share the same architecture. They fold around the L-domain of the mature enzyme, as shown for the propeptide of cathepsin B (Figure 3a) ([98], PDB code 3PBH, Table 4). Exceptionally, the papain-like enzyme is shown in an orientation in which the active site cleft runs from left to right (standard view is from bottom to the top), which brings the so-called R- and L-domains to the bottom and top of the image. They wrap around the R-domain of the mature part of the enzyme. They build an N-terminal, predominantly a helical domain of various sizes, which binds to the surface of the L-domain of the enzyme on the prime side (left in the figure) and enters the active site cleft with an α-helix ending above the pair of catalytic cysteine and histidine residues (colored red). Then, the chain continues in the direction opposite to substrate binding along the active site and turns down where it joins with the enzyme’s N-terminus. Several structures of proenzymes of papain-like proteases (Table 4) have been determined (procathepsins B ([109], PDB code 1MIR; [98], PDB code 3PBH), L ([106], PDB code 1CJL, 1CS8), H ([113], PDB code 6CZK/6CZS), K ([110], PDB code 7PCK; [111], PDB code 1BY8), and S ([112], PDB code 2C0Y), and propapain ([107], PDB code 3TNX)); among them, the propeptide of procathepsin X appears the shortest ([101], PDB code 1DEU). Table 4 shows the zymogens.

Its N-terminal domain is composed of a short peptide only, for which the reactive site attachment is strengthened by a disulfide formed between the reactive site C31 and propeptide C10P. In contrast, the propeptide of cathepsin L has the largest N-terminal domain, composed of 96 residues. The role of N-terminal domains appears to be the same; they anchor the propetide in the primed side of the structure to enable its stretched binding along the active site cleft to the enzyme’s N-terminus.

### 4.2. Cystatin E and Macrocypins

Cystatin E and macrocypins, such as macrocypins 1 and 3, can, in addition to papain-like proteases, also inhibit legumain (known also as asparagine endopeptidase or AEP) and macrocypin 4, and even the serine protease trypsin [49]. Among these, only the crystal structure of the complex between cystatin E and legumain has been determined (Figure 3b) ([42], PDB code 4N6N). In the complex, cystain E makes contact with legumain with two loops. The first, called the reactive center loop, encompasses residues from G37 to I41, which bind across the reactive site in a substrate-like manner with the N39 side chain binding in the pocket S1, which specifically recognizes asparagine residues and, under acidic conditions, also accepts aspartic residues. The second loop from D72 to Q96, called the exosite loop, includes residues from R74 to D81, which make contact in the region of the primed substrate binding sites. Because the reactive site loop binds as a substrate, Dall et al. [42] investigated the possible cleavage and observed that cystatin was indeed cleaved after N39 and that, over time, the ratio between the cleaved and uncleaved cystatin remained constant. On the basis of the subsequent analysis, which included chemical modification of the reactive site C189 with S-methyl methanethiosulfonate, the authors arrived at the conclusion that, at neutral pH, legumain behaves as a ligase and C189 is not involved in this reaction. They assigned the catalytic activity to a different catalytic center, which they assigned in an unorthodox manner to succinate 147, a chemical modification of D147, clearly recognizable in the electron density map. In a follow-up theoretical work, they confirmed the initial idea that cysteine is not involved in the ligase reaction, however, the catalytic center was assigned to H148 [137], and succinate 147 carbonyl was used to stabilize the side chain of H148. The ligase activity of cysteine proteases as a consequence of the pH of the media may be a common phenomenon. It was also observed for papain at pH above 9 [138] and cathepsin C at neutral pH [139,140].

### 4.3. Calpastatin

Calpastatin is a highly selective inhibitor of calpains, which are Ca^2+^-dependent multidomain cysteine proteases with the catalytic domain that shares some resemblance to the papain fold. Calpains are involved in a number of processes including cell migration, cell death, insulin secretion to synaptic function, and muscle homeostasis [141,142,143,144,145], whereas under pathological conditions they have been linked to cell death by necrosis induced by stroke [91], neuronal injury and perhaps Alzheimer’s disease [91,146], heart disease [146], cataract formation [91,146], type 2 diabetes [91,146,147], cancer, and limb-girdle muscular dystrophy type 2A [91,146,147].

The crystal structure of the complex between m-calpain and the first repeat of calpastatin truncated to the residues from 119 to 238 ([99], PDB code 3DF0, Table 5) reveals that calpastatin is a polypetide that adopts a three-dimensional structure in the presence of its target, calcium-activated m-calpain (Figure 3c). Parts of the chain remained unstructured even after binding to calpain. In Figure 3c, calpastatin is presented as a surface model because the chain trace of the calpastatin coil appears too small to be resolved. The calpastatin chain binds in the active site cleft in the direction of the substrate with L175 filling the specificity pocket S1 (please note that we do not follow the authors numbering from the publication, but instead follow the numbering of residues in the PDB file).

**Table 5:** List of the available structures of the complexes between protein inhibitors and cysteine proteases: calpastatins, securing, and designed ankyrin repeat proteins (DARPins).

	Protein Inhibitor			Cysteine Protease			
MEROPS ID	Name	Organism	MEROPS ID	Name	Organism	PDB ID	Reference
I27	**Calpastatin family**						
I27.001	calpastatin	*Rattus norvegicus*	C02.002	calpain-2	*Rattus norvegicus*	3DF0	[99]
I27.001	calpastatin	*Rattus norvegicus*	C02.002	calpain-2	*Rattus norvegicus*	3BOW	[148]
	securin	*Saccharomyces cerevisiae S288C*	C50.001	separin	*Saccharomyces cerevisiae S288C*	5U1S/5U1T	[100]
	Interactor of FizzY protein	*Caenorhabditis elegans*	C50.004	separase	*Caenorhabditis elegans*	5MZ6	[149]
	DARPin	synthetic construct	C14.006	Caspase-2	*Homo sapiens*	2P2C	[150]
	DARPin-3.4	synthetic construct	C14.003	Caspase-3	*Homo sapiens*	2XZD	[151]
	DARPin C7_16	synthetic construct	C14.004	Caspase-7	*Homo sapiens*	4JB8	[152]
	DARPin D7.18	synthetic construct	C14.004	Caspase-7	*Homo sapiens*	4LSZ	[153]
	DARPin 8h6	synthetic construct	C01.060	Cathepsin B	*Homo sapiens*	5MBM	[154]
	DARPin 81	synthetic construct	C01.060	Cathepsin B	*Homo sapiens*	5MBL	[154]

However, the chain then turns away from the catalytic residues, forming a cross-over of the reactive site with the IKEGT sequence called “loop out”, colored red in Figure 3c, and only thereafter following the active site cleft with I182 and a pair of proline residues that lie at the N-terminus of a helix. The helix just beyond the active site cleft in the primed side is reminiscent of cathepsin propeptide structures; however, their chains run in the opposite directions so that the helix N-terminus of calpastatin, rather than the C-terminus, is positioned near the active site. The exact sequence and length of the loop out region appears crucial for inhibition because the deletion mutant of K178 abolishes any inhibition.

### 4.4. Securin

Securin inhibits separase, a protease involved in separation of sister chromatids during chromosome segregation during somatic cell division at mitosis and meiosis [155,156,157,158,159,160,161]. The crystal structure ([100], PDB code 5ULS, 5ULT) and the electron microscopy (EM) structure of intermediate resolution ([149], PDB code 5MZ6) of the complex were determined almost simultaneously (Table 5). Due to having a substantially more complete model and more accurate insight, we have shown the crystal structure of *Saccharomyces cerevisiae* securin (Figure 3d). Separases are large four-domain proteins with chains longer than 1600 residues. The C-terminal domain is catalytic. There was a disagreement between Luo et al. [100] and Boland et al. [149] regarding whether separase is a caspase-like enzyme. FatCat [162] found that 146 residues could be aligned between the human caspase-3 and the catalytic domain of *S. cervisiae* separase with an root mean square deviation (RMSD) of 3.1 Å and 8% sequence identities. Hence, folds are superimposable. In addition, upon visual inspection, a high similarity of the folds is clearly recognizable. However, a number of secondary structure elements do not share deviations within the 3 Å of RMSD. Moreover, separase substrate specificity differs from the specificity of caspases. In contrast to caspases, separase cleave substrates after an arginine at P1, which is, however, characteristic of metacaspases, and glutamate at P4 [156,161,163]. In addition, loops surrounding the active site cleft provide a different means of entry to the substrate and potential inhibitors. Hence, in addition to the difference in molecular size and the number of domains, separases and caspases belong to two different protease families according to MEROPS classification [15]. The structures of the complex reveal that securin—which starts in the structure with M257 and runs to E361, and is unstructured in the naked form—binds along and around all four domains of separase, including the whole active site of the enzyme. No own secondary structure motifs stabilize its conformation, which relies completely on binding to discontinuous grooves of its target. Consequently, a substantial part of the securin structure in the complex remains unstructured. When preparing Figure 3d, we decided to show securin in a similar fashion to that of calpastatin in Figure 3c, that is, as a surface rather than a coil, due to its small size in comparison to the large separase structure. The region P263 to R265 is colored red to indicate the position of the reactive site. Although the securin chain binds in the active site cleft in the direction of a substrate, it is not a substrate analogue. S3 is filled with I261, however, the S1 pocket is instead filled with arginine covered with a proline residue P263, conformational rigidity of which likely makes securin non-cleavable, and then turns away from the catalytic pair C2110–H2083 and bypasses it. Securin regulates separase activity; however, it interacts with all domains, including those carrying no proteolytic activity, and hence its regulatory function is far more complex than the simple regulation of separase proteolytic activity.

## 5. Mechanisms of Inhibition

The brief analysis presented here suggests that the protein fold does not present a major obstacle for the evolution of a protease inhibitor. Numerous folds are adopted to inhibit proteases. Simple and small folds exist, such as cysteine cathepsins’ propeptide domains based on the α-helical folds, cystatins and p41 fragments using a β-sheet combined with an α-helix, as well as ICPs from *Plasmodium*, staphostatin, chagasin, clitocypins and macrocypins, and the exclusion domain of cathepsin C [119] using β-barrels of various strand numbers and architectures. More complex folds also exist that contain several motifs that combine α-helical and β-sheet elements, such as in serpin CrmA, p35, and XIAP. Some of the inhibitors are embedded in larger protein chains, such as the p41 fragment, and some appear as multiple repeats including kininogens [10], equistatin [58], and calpastatins ([99]). In addition, peptide-like inhibitors exist without a folding pattern, such as calpastatin and securin, which appear to fold only in contact with their target protease. Our understanding of protein inhibitor protease interactions follows the understanding of the substrate enzyme interaction models. The first model to explain the match between a substrate and an enzyme, introduced by Fischer, used the “lock and key” analogy [164]. In the 1950s, this model was enriched by Koshland with the ”induced fit” theory [165]. More recently “conformational selection” was introduced to describe the dynamic of binding events [166]. From the behavior of partially or completely unfolded inhibitors, it appears that their dynamic surpasses the extent of dynamics in the conformational selection model because some protease inhibitors appear to lack discernable conformations in the pre-bound state.

Our survey of the structures of the majority of cysteine protease protein inhibitors in complexes with cysteine proteases shows that the diversity of inhibition mechanisms appears to be unlimited. It appears as if nature has found numerous means of successively overcome almost any starting fold. Thus, it is to be expected that the folds first observed in protein inhibitors, such as cystatins and serpins, may be used in proteins exhibiting other physiological roles and functions, such as monellin, a sweet-tasting protein with the cystatin fold (reviewed in [167]), or the non-histone architectural protein myeloid and erythroid nuclear termination stage-specific protein (MENT), which participates in DNA and chromatin condensation [168]. The challenge is whether we can do the same. Does science provide enough insight, understanding, and tools to enable us to design protein inhibitors of proteases for medical, agricultural, and industrial uses?

**Figure 4:**
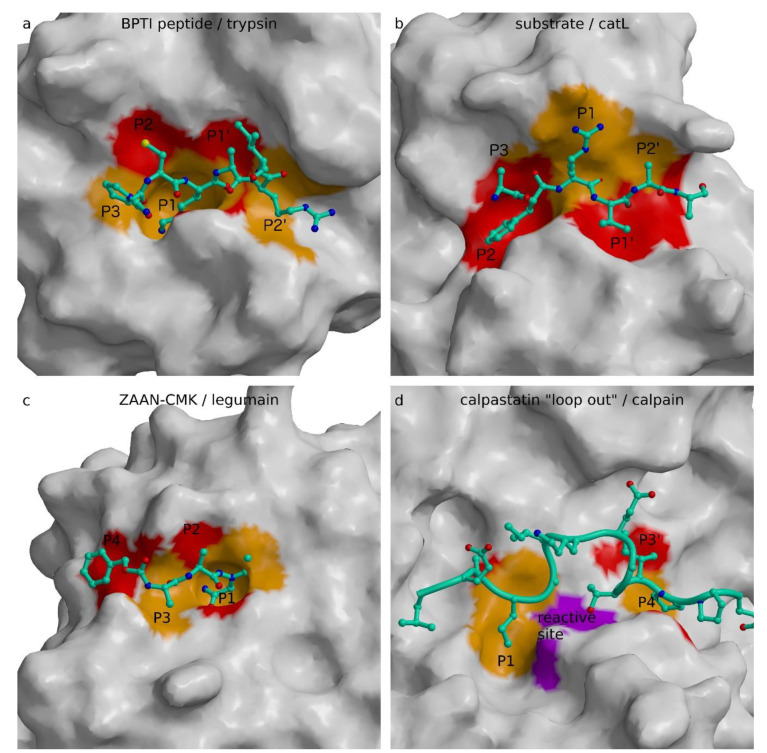
Substrate and substrate-like binding. Peptidyl substrates with their positions marked are shown as ball (nitrogen: blue, oxygens: red, carbons: cyan) and stick (cyan) models on the background of the protease surface. The surface is white with the exception of the substrate binding sites S3, S1, and S2′, respectively corresponding to substrate positions P3, P1, and P2′ colored orange, and S4, S2, and S1′ respectively corresponding to substrate positions P4, P2, and P1′ colored red. The figure was prepared using MAIN [35] and rendered with Raster3d [36]. (**a**) Canonical conformation of BPTI peptide bound to trypsin ([24], PDB code 2TGP). BPTI peptide is shown as a ball and stick model on the background of trypsin structure shown in white, with the exception of the substrate-binding sites surface from S3 to S3′ colored alternatively red and orange. (**b**) Substrate model bound to cathepsin L. The cathepsin L model was used from a previous study [11]. (**c**) Peptidyl inhibitor bound to legumain ([170], PDB code 4AWB). Z-Ala-Ala-Asn (ZAAN) binds to the non-prime region of the active site cleft. (**d**) Calpastatin loop out region bound to calpain-m ([99], PDB code 3DF0). The 172–185 region of calpastatin is shown as a coil for the main chain trace, and a ball and stick model for side chains on the background of the protease surface. The calpain surface was generated with the residues from S241 to V253, I260, and Q261 excluded to enable the view in the active site cleft. The surface of the reactive site residues C105S and H262 is purple.

To provide insight, we revisited the review of Bode and Huber of the interactions of natural protein protease inhibitors [5]. This seminal work suggested that the era of the substrate-like canonical serine protease inhibitors of different folds, and the same active site binding geometry and product-like inhibition of carboxypeptidase inhibitor, ended with complexes of stefin B with papain [21] and hirudin with thrombin ([169], PDB code 3HTC), which revealed non-substrate-like interactions. In addition, protein inhibitors of cysteine proteases can be divided into two groups: those mimicking a protease substrate and those that do not. To demonstrate the requirements imposed by the structure of the active site cleft, we prepared Figure 4 with the canonical region of the BPTI in the complex with trypsin to compare it with the modeled substrate in the active site of cathepsin L, with the substrate analogue, inhibitor Z-Ala-Ala-Asn-chloromethyketone (ZAAN-CMK), in the complex with legumain, and the loop out construct of calpastatin. The canonical conformation of BPTI and the substrate model of cathepsin L, as well as ZAAN-CMK, all bind in an extended conformation along the active site cleft in the direction of the N- to C-peptidyl termini running from left to right. The up and down directions of the side chains clearly demonstrate the opposite patterns. Using the same alternate red and orange coloring for the surface of substrate-binding sites from S3 to S2′, one can expose a reverse color pattern between the active sites of trypsin (Figure 4a) and legumain (Figure 4c) on the one side and cathepsin L (Figure 4b) on the other. Whereas the trypsin and legumain upper parts of the surfaces are red and lower orange, the opposite coloring pattern is present in the cathepsin L surface. The orange S1 pocket pointing inwards in trypsin is the structural feature that dominates its arginine/lysine specificity, similar to the S1 pocket in legumain with asparagine/aspartate specificity, whereas the S1 in cathepsin L has no pocket—it is merely a surface to which substrate side chains can attach from a side. Hence, papain-like proteases provide significantly less structural restraints and enable broader selectivity of residues at P1. There is one important consequence of the shape and position of the S1 binding site, namely, the presence of the S1 pocket that requires the substrate P1 residue side chain to point away from the substrate surface—that is, towards the inside of a protein substrate or towards the solvent in the case of structural restraints free of peptide chains. This suggests that cathepsin L-like substrates must exhibit considerable flexibility to be able to adopt their binding geometry to the active site of cathepsin L–like protease. As a consequence, inhibitors of papain-like cysteine proteases cannot mimic a “canonical” substrate-like geometry because it embeds flexibility of the putative “canonical” region. Hence, their inhibitors also cannot mimic a substrate without being cleaved. We are thereby compelled to suggest that protein inhibitors targeting papain-like proteases in a substrate-like manner do not exist. Serpins are no exception to this rule, because they do not bind their target papain-like protease in a stable canonical conformation, but are essentially suicide substrates that, rather than remaining bound, use a flexible reactive site loop to pull out a part of catalytic site before the reaction can be completed, and remain loosely attached to the enzyme outside the immediate active site region, as indicated by the structure of the trypsin anti-trypsin complex [124]. Serpins are a cross-class type of protease inhibitors because they can pull out the catalytic residue using the ester bond-formed nucleophilic residues, such as serine and cysteine. Because ester bonds attached to the enzyme cannot be formed with the solvent molecule, which plays the role of a nucleophile in aspartic/glutamic and metalloproteases, we are therefore unlikely to find a serpin inhibiting these two classes of proteases.

Overall, the division of proteases according to those with the S1 binding site shaped as a pocket, and those with the S1 binding site loosely formed at the surface, enables identification of the families of proteases that can be targeted with protein inhibitors that bind in the active site cleft in a substrate-like manner. Among the proteases inspected here, only papain-like cysteine proteases do not possess an S1 binding pocket. This explains why protein inhibitors apply combinations of loops or chain-loop constructs to occlude the reactive site, while binding into the active site cleft of their targets. However, when the concept of spanning the active site by a peptidyl chain is applied, as in the case of propeptides, the chain can only run in the direction opposite to the substrate binding.

For inhibition of the families of proteases that possess S1 shaped as a specific binding pocket, there appear to be fewer restraints in the concept. It appears that the S1 binding pocket implies smaller dynamics of a peptidyl substrate, and thus inhibitors can implement a single chain that spans the active site and still bypass the catalytic site. Among these are solutions such as “loop out” in calpastatin (Figure 4d) and specific uncleavable sequences with residues at P1, such as glycine in staphostatin B and proline in securin. The mechanism of cystatin inhibition of legumain is the closest to the “canonical” conformation of serine proteases because it applies the substrate-like binding, however, in combination with a loop. In concept, this is similar to the p35 inhibition mechanism of caspases. P35 uses residue D87, which remains bound to the enzyme after cleavage. This concept is, in turn, similar to the Nlef inhibition mechanism. Nlef binds with its last four C-terminal residues in the non-primed part of the caspase active site cleft and provides the negatively charged C-terminal G199 to bind in the S1 pocket.

## 6. Concluding Remarks

In the age of protein engineering, we would like to go beyond repurposing natural design. We would like to apply our knowledge and understanding to the design of protein inhibitors that regulate, mark, or block the activity of proteases in biological systems. We found no inhibitors in clinical trials or in use as a drug (WHO International Clinical Trials Registry Platform ICTRP: https://www.who.int/ictrp/en/; ClinicalTrials.gov: https://clinicaltrials.gov/ct2/results?cond=COVID-19). Of interest, however, Novartis developed a small molecule inhibitor, LCL-161, a second mitochondria-derived activator of caspase (SMAC) mimetic, which binds to XIAP and loosens the binding of XIAP to caspase-9, thereby promoting cell apoptosis [171]. LCL-161 is in clinical phase II for the treatment of breast cancer. We are still at the beginning of the “de novo” design of proteins; however, we are capable of adopting existing concepts found in nature. For substrate-like inhibitors that bind to the non-primed part of the active site cleft, the simplest approach appears to be to tap the substrate specificity with high throughput screens such as [172], and build these sequences into the inhibitor of interest. Serpins, baculovirus p35, macrocypins, Nlef and likely others, including BPTI, could be used to engineer the desired specificity. Serpins appear to be an ideal scaffold because their flexibility and unique mechanism likely address every protease class that contains a reactive site nucleophile capable of forming ester intermediates. For example, Whisstock and his team engineered α1-antitrypsin to inhibit cysteine cathepsins L, V, and K [173]. Moreover, the scaffold of stefins has also been used to develop a targeted drug delivery system [174] and to generate binders for proteins not related to cysteine proteases [175,176]. The binding loop of cystatins was engineered in plant cyclotide, a 35-residues-long cyclic peptide cross-linked with three disulfide bonds called McoTI-II, and reached μM binding [177]. However, to engineer tight binding inhibitors of papain-like proteases (in addition to serpins) on the basis of non-substrate-like approaches, generic tools should be utilized, including antibodies or structural repeats such as designed ankyrin repeat proteins (DARPins), which were engineered to inhibit cathepsin B in the pM range. These were found to have a substantially higher affinity than those shown for any of endogenous inhibitors [154]. One of the most studied protein serine protease inhibitors, BPTI, under the name aprotinin, has been in and out of and again in use in surgery to slow fibrinolysis (blood clot degradation) during complex surgical procedures. Recently, reexamination of its potential in the treatment of pancreatitis was encouraged by analysis of previous studies, which argued that previous clinical studies lacked an adequate biochemical background [178]. It appears that the technology and knowledge of protein inhibitor engineering has not yet reached a level comparable to that of small molecule design. One obstacle is the specificity of binding. Protein inhibitors do not inhibit a single protease molecule, but usually bind to a group of related enzymes with different affinities. Nevertheless, we believe that the potential exists. Most mechanistic studies mentioned in this review targeted the main interaction regions within the vicinity of the reactive sites. Only a few studies, such as the study of interactions between the p41 fragment and cysteine cathepsins [49], systematically addressed the binding affinity of an inhibitor against a group of related enzymes using site-directed mutagenesis. We hope that the analysis and understanding of interactions between protein inhibitors and their protease targets presented here may encourage and assist in the application of protein inhibitors in medical, agricultural, and industrial applications.

## Data Availability

Not applicable.

## Appendix A

**Table A1:** Compiled kinetic constants Ki for the comparison of inhibitors potency presented as ranges of values reported by the researchers in the publications. (**a**) Ranges of binding constants for cathepsin inhibitors Ki (nM). (**b**) Range of binding constants for caspase inhibitors Ki (nM). (**c**) Ranges of binding constants for papain and similar protease inhibitors Ki (nM). (**d**) Ranges of binding constants for the small group of protease inhibitors Ki (nM).

(**a**)												
**Inhibitors of Cathepsins Ki (nM)**		**Cathepsin B**		**Cathepsin L**	**Cathepsin H**	**Cathepsin K**	**Cathepsin S**	**Cathepsin V**	**Cathepsin C**	**Cathepsin X**	**Cathepsin F**	**References**
Stefin A		1.79->10,000		0.0034–1.3	0.069–2400		0.053–0.27		1.1–5340	1.7		[34,56,174,179,180]
Stefin B		73		0.23–13	0.14–930					>250		[11,34,179,181]
Cystatin		101		11.5	0.63	25.4						[32]
Cystatin A		8.2		1.3	0.31		0.05		33			[6,30]
Cystatin B		16–73		0.23	0.58		0.07		0.23			[6,30,46]
Cystatin C		0.25–18,000		<0.02	<0.005–1.8		0.008		0.5–3.5	12		[6,23,31,34,38,179,182,183,184]
Cystatin Chicken		0.07–4		0.019	0.064				0.35	15		[6,27,31,98,179]
Cystatin D		>1000		5.8–25	7.5–18		0.24					[11,30,38,182,183]
Cystatin E		32										[182]
Cystatin E/M		31–32										[38,183]
Cystatin F		>1000		0.31								[38,183]
Cystatin SN		19										[30]
Kininogens HMW		400		0.019–0.109	1.1							[6,11,27,183]
Kininogens LMW		600		0.017–0.048	0.72–1.2				>130	>1000		[6,30,179,185]
Kininogen segment 1				>100								[6]
Kininogen segment 2				0.14								[6]
Kininogen segment 3				0.005								[6]
Falstatin				0.032	0.052	0.025						[44]
Chagasin		0.35–100		0.039–0.35	15	2						[32,37,38,46,47,51]
p41 fragment		>1000		0.00149–0.01	5.3	0.09	0.27–208	0.0072		>1000	0.51	[11,55,186]
Clitocypin		>1000		0.02–0.03		0.02–0.03	2.2–3.2	0.08–1.9				[11,40,49,55,70,186]
Macrocypin		515		3.81	370	4.5	47.1	8.5–12.6				[40,49]
Macrocypin 1		490		0.64	100	170	23.1	0.69–12.5				[40,49]
Macrocypin 3		>1000		0.31	24	17.5	5.1	0.45–3.43				[40,49]
Macrocypin 4		125		2.76	32	21.8	6.3	1.44–10.2				[40,49]
Serpins				35.85–71.06								[81]
Equistatin		1.4		0.051								[58]
Leupeptin		0.37		0.52	3.2	0.64						[32]
Saxiphilin		1.67		0.02								[58]
Cysteine proteinase inhibitor		15.8–281		0.062–3.607								[64,185,187]
Propeptide Cathepsin K		>600		3.6		5.5	6.3					[136]
Propeptide Cathepsin L		>600		0.12		0.27	65					[136]
Propeptide Cathepsin S		>600		0.46		7	7.6					[136]
Propeptide Cathepsin B		0.4–64										[134]
Propeptide Cruzipain				2.05				5.18			0.032	[188]
(**b**)												
**Inhibitors of Caspases Ki (nM)**	**Caspase-1**	**Caspase-2**	**Caspase-3**	**Caspase-4**	**Caspase-6**	**Caspase-5**	**Caspase-7**	**Caspase-8**	**Caspase-9**	**Caspase-10**	**Pro-Caspase 7**	**References**
XIAP			<0.4->1000				<0.05->1000					[102]
XIAP BIR2 BIR3			3				1					[189,190]
XIAP BIR3			>1000				>1000		13			[189,190]
XIAP BIR1			>1000				>1000					[189]
XIAP BIR1 BIR2			3				1					[189]
XIAP BIR2			<0.4->1000				<0.05->1000					[83,91,102,189]
MLBIR									3200			[190]
MLBIR-Q									<<10–4500			[190]
MLXBIR3									<<10–960			[190]
p35	9		0.11–34,000		0.38		1.8	0.48–400		7		[85,114,191]
Serpin Darpin AR_F8		0.29										[150]
Serpin DARPin D3.4			3.49–16.8									[151]
Serpin DARPin D3.8			6.7									[151]
Serpin DARPins D7.18							144				295	[153]
Serpin DARPins D7.43							24.7				32.2	[153]
DARPin							33–597					[152]
Serpin SERP2	0.1–0.2							0.5–7.4		1.3–200		[130]
CrmA	0.01	>10,000	500–1600	1.1	110–1300	<0.1	>10,000	<0.34	<2.3	17		[129,130,131,132]
Falstatin			376					80				[44]
(**c**)												
**Inhibitors of Proteases Ki (nM)**		**Papain**	**Calpain**	**Calpain-1**	**Falcipain**	**Falcipain-2**	**Falcipain-3**	**Ficin**	**Cruzi-pain**	**Legu-main**	**Actini-din**	**References**
Stefin A		0.019–87.20							0.021–0.072			[174,179,180,192]
Stefin B		0.12–110							0.06			[11,34,179,181,192]
Cystatin						6.5	100					[32]
Cystatin A		0.019	>10,000									[6,7,30]
Cystatin B		0.034–0.12	>10,000									[30,46]
Cystatin C		0.00001–11	>10,000					0.0013–29	0.0048–0.014		19-≥40,000	[6,7,23,38,179,182,184,192,193]
Stefin A		0.019							0.0072			[11]
Stefin B		0.12							0.06			[11]
Cystatin Chicken		0.00006–0.005	>10,000					0.00005	0.001–0.0028			[6,27,31,179,192]
Cystatin C		0.00001–1.7						23–29	0.014		1100–40,000	[11,23]
Cystatin D		0.9–1.2										[7,11,38,182]
Cystatin E		0.39										[182]
Cystatin E/M		0.39–0.46										[38,183]
Cystatin F		1.1										[38,183]
Cystatin S		108										[7,30]
Cystatin SA		0.32										[7,30]
Cystatin SN		0.016										[7,30]
Kininogens HMW		0.02										[6,185]
Kininogens LMW		0.015–0.017	1						0.041			[6,30,179,185,192]
Kininogen segment 1		>100	>100									[6]
Kininogen segment 2		0.083	1									[6]
Kininogen segment 3		0.03	>100									[6]
Falstatin		53.5		0.196		0.021–0.045	0.223					[44]
Serpin B3		220.48–293.49				277.27–338.11						[81]
Chagasins		0.013–0.036			1.7	4.8	0.062		0.0067–0.095			[32,37,38,46,47,51]
p41 fragment		1.4							0.058			[55,186]
Clitocypin		2.5–6.2								7.1–21.5		[40,49,70]
Macrocypin		5.04								110		[40,49]
Macrocypin 1		0.95								3.38		[40,49]
Macrocypin 3		0.12								9.17		[40,49]
Macrocypin 4		0.19								2.86->1000		[40,49]
205-residue N-terminal prodomain												[105]
Equistatin		0.57->1000										[58]
Leupeptin						0.2	0.3					[32]
Phosphonoformate												[194]
Saxiphilin		1.72										[62]
Cysteine proteinase inhibitor		0.034–0.35				8.7–37.1						[37,64,185,187]
Propeptide Cathepsin K		>600										[136]
Propeptide Cathepsin L		>600										[136]
Propeptide Cathepsin S												[136]
Propeptide Cruzipain									0.018–0.2637			[188]
Propeptide Cathepsin B		2800–5600										[134]
(**d**)												
**Inhibitors of Proteases Ki (nM)**		**Vivapain-3**	**Berghepain-2**	**Vinckepain-2**	**Knowlepain-2**	**Gingipain**	**FosA**	***Leishmainia mexicana* CPB2.8∆CTE**		**Brucipain**	**Thrombin**	**References**
Falstatin		0.065	0.048	0.15	0.078							[44]
205-residue N-terminal prodomain						6.2						[105]
Phosphonoformate							400					[194]
ICP								0.071–0.495				[37,64,187]
Propeptide cruzipain										0.0163		[188]
Hirudin											0.00001–0.01	[169]
